# *Ligia italica* (Isopoda, Oniscidea) as Bioindicator of Mercury Pollution of Marine Rocky Coasts

**DOI:** 10.1371/journal.pone.0058548

**Published:** 2013-03-05

**Authors:** Guglielmo Longo, Michelanna Trovato, Veronica Mazzei, Margherita Ferrante, Gea Oliveri Conti

**Affiliations:** 1 Dipartimento di Scienze Biologiche, Geologiche e Ambientali, Università di Catania, Catania, Italy; 2 Dipartimento di Anatomia, Biologia e Genetica, Medicina Legale, Neuroscienze, Patologia Diagnostica, Igiene e Sanità Pubblica “G. F. Ingrassia”, Università di Catania, Catania, Italy; Université du Québec à Rimouski, Canada

## Abstract

In this study, we evaluated the possible role of *Ligia italica* as a bioindicator for the monitoring of heavy metals pollution in the suppralittoral zone of marine rocky coasts. Between 2004 and 2011 specimens of *L. italica* were collected along the Eastern Sicilian coasts from sites known for their high pollution levels as they are near to an area where in September 2001 a refinery plant discharged into the sea some waste containing Hg. Other specimens were collected from the Vendicari Natural Reserve located about 30 miles from the polluted sites and used as control area. On a consistent number of animals, the concentration *in toto* of As, Cd, Cr, Hg, Ni, Pb, V, was determined by Atomic Absorption Spectrometry. On other animals, investigations were carried out in order to check for ultrastructural alterations of the hepatopancreas, that is the main metals storage organ in isopods. Results revealed the presence, in the animals collected in 2004 from the polluted sites, of considerable concentrations of Hg and of lower concentrations of other metals such as As, Pb and V. The Hg bioaccumulation resulted in remarkable ultrastructural alterations of the two cellular types (B and S cells) in the epithelium of the hepatopancreas. Surprisingly, a moderate amount of Hg was also found in specimens collected in 2004 from the Vendicari Natural Reserve, proving that the Hg pollution can also spread many miles away. Animals collected from the polluted sites in the following years showed a progressively decreasing Hg content, reaching very low levels in those from the last sampling. Also, the ultrastructural alterations found in the hepatopancreas of the animals from the last sample were quite irrelevant. In conclusion, *Ligia italica* can represent a good bioindicator and the ultrastructure of the hepatopancreas could be used as ultrastructural biomarker of heavy metals pollution in the supralittoral zones.

## Introduction

The coast is the frontier between land and sea. On the rocky shores the supralittoral zone is the emerged area regularly reached by the sea spray and/or splash. In the supralittoral zone of the sea rocky coasts the dynamic influence of the marine water interacts with the terrestrial environment. The supralittoral, for this reason, is an ecotone particularly subject to pollution because it receives contaminants coming from both environments [1]–[2]; it is characterized by strong thermal range, intense light radiation and significant changes in salinity, depending on evaporation and rainwater inputs (trends in rainfall or rainwater regime). Therefore, its wildlife shows features that exhibit affinity with both environments. The most numerous animal group is represented by crustaceans, particularly, by Isopoda, Amphipoda and Decapoda [3]–[4].

In the last years, the development of sensible techniques and sophisticated assays has improved the environmental monitoring programs by using valid bioindicators of marine ecosystems, particularly for monitoring heavy metals in the sea water [5]–[8]. A biological indicator is a organism used to monitor the environmental quality of a particular ecosystem that provides quantitative information on the quality of the environment around it. Therefore, a good bioindicator will indicate the presence and the amount of a pollutant and will provide additional information about the intensity of its exposure [9]–[11]. A bioindicator specie is considered valid if some basic characteristics are satisfied, in particular: accessibility, bio-ecological suitability, reliability and representativeness.

The marine heavy metals pollution is an issue for many Eastern Sicilian coasts where several industrial and petrochemical plants have discharged massive quantities of polluting gases, organic contaminants and heavy metals, thus causing dramatic levels of pollutants in air, water and land with consequent harmful outcome to human health [12]–[15]. In 2001, for example, one refinery plant dumped into the Sicilian sea a considerable amount of mercury (Hg) and other polluting heavy metals; in fact, analyses requested by the Public prosecutor's office of Syracuse demonstrated that the amount of Hg present in the sea was twenty thousand times higher than the upper limit established by law. The consequences of contamination by Hg [16]–[17], and by other metals as well, are known for many years, as they pose a worldwide problem [12]–[14]. However, despite the seriousness of that episode of pollution, until now, the knowledge of the environmental status is lacking because studies aimed at evaluating its effects on biological communities present in the concerned area are numerically very poor, probably due also to the lack of a validated biondicator for studying the supralittoral zone of the rocky coasts.

*Ligia italica* Fabricius 1798 (one of many coastal *Ligia*) is an oniscidean isopod particularly abundant in this Sicilian ecotone and it is being closely bound to the marine environment from which leaves only temporarily and only for few meters [4]; [7]. 'The low vagility and high levels of isolation observed among populations of coastal *Ligia* make this isopod a potential good bioindicator, free from cross contamination, as they appear to be highly constrained throughout their life cycle to the same rocky beach [4]. The effectiveness of Isopoda as excellent bioindicators and bioaccumulators of heavy metals in biomonitoring programs is claimed by scientific literature [4]; [18]–[27]. The main metal storage organ in isopods is the hepatopancreas [28]–[29]. Specifically, Hg accumulates mainly in the exoskeleton while cadmium, lead and chromium can accumulate preferably into the hepatopancreas [29]. In the light of these considerations, it is extremely important to verify the potential for bioaccumulation and biological responses of *L. italica* Fabricius 1798 in order to assess their health status and, indirectly, to evaluate the quality of the environment in which they live.

In the present research we evaluated the possible role of *Ligia italica*, as bioaccumulator and bioindicator, in monitoring Hg and other heavy metals pollution in the supralittoral zone of marine rocky coasts. The validation of this species as bioindicator has been carried out by calculating the amount of Hg and other heavy metals, such as arsenic (As), cadmium (Cd), chromium (Cr), nickel (Ni), lead (Pb), and vanadium (V), in animals in toto and by observing their effects on hepatopancreas ultrastructure.

## Materials and Methods

**Figure 1:**
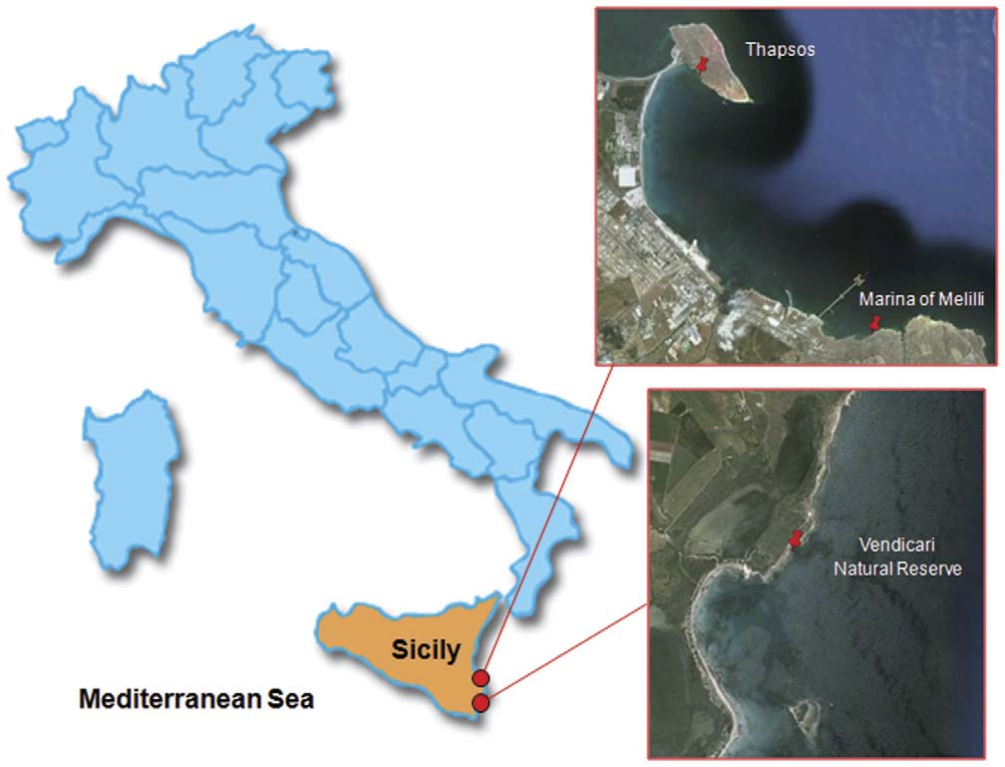
Map of sampling sites.

The research was carried out on a consistent number of sexually mature male and female *L. italica*, samples were collected twice in 2004, 2007 and 2011 from three different sites of the district of Syracuse (Sicily): Thapsos (Penisola Magnisi), along the rocky shore in front of the petrochemical pole (where in 2001 the discharge of a large amount of Hg was registered); Marina di Melilli, another very polluted coastline near Thapsos, and the Vendicari Natural Reserve (see Fig. 1). We chose the last site, being non-polluted, because we wanted to utilize it for collection of control samples. In order to calculate metals concentrations, from each of the three research sites, we took three groups of ten animals (five males and five females); they were weighed, beheaded and immediately immersed in 10 ml vials containing 3 ml of HNO_3_ (Suprapur) +1 ml of H_2_O_2_ (mineralization solution). Analysis were carried out in the Laboratory of Environmental and Food Hygiene of the Hygiene and Public Health Department “*G. F. Ingrassia*”, University of Catania, through the following steps:

1. the digestion solution was adjusted to a final volume of 6 ml of HNO_3_ and 1 ml of H_2_O_2_;
2. samples were mineralized in a Milestone 1200 microwave digestor, in teflon dedicated vessels, using the specific program;
3. then, samples were transferred into 10 ml flasks and were brought to volume with ultrapure metal free water for determination of As, Cd, Cr, Ni, Pb, V, by the atomic absorption spectrometer (AAS) Perkin Elmer Analyst 800.

For Hg determination, we used the FIAS-AAS (Cold vapour Technique or Hydride Generation System) with the same Analyst 800. The instrument was properly calibrated for each metal by means of certified Perkin Elmer calibration standards. The values of metals contents were reported in μg g^−1^ dry weight. Statistical analysis was performed using the Kruskal-Wallis test on SPSS-18.0 software.

The ultrastructural investigations were carried out on the hepatopancreas, the organ primarily involved in the bioaccumulation of heavy metals. Some males and females were beheaded and immediately immersed in Ringer's saline solution modified for land isopods acc. Legrand cited by Besse [30]; then the hepatopancreas were removed. Fixation was carried out through 2.5% glutaraldehyde in 0.1 M phosphate buffer, pH 7.3 for 4 h at 4°C; after repeated washing in the same buffer the specimens were post fixed in 1% OsO_4_, in the same buffer, for 1 h at room temperature. The samples were dehydrated in ethanol followed by propylene oxide and embedded in Embed 812 (EMS). For light microscopy, semi-thin sections were cut on a Ultracut Leica ultramicrotome with diamond blades, then they were stained with 0.5% blue toluidine in 0.1 M phosphate buffer, pH 7.3 or with the polychromatic staining method of Sato and Shamoto [31]. For electron transmission microscopy, ultra-thin sections were collected on Cu/Rh grids of 200 mesh, were stained with uranyl acetate and lead citrate acc. Reynolds [32] and examined in a Philips CM 10 electron microscope, at 60 or 80 kV.

## Results

### Metal concentration

**Figure 2:**
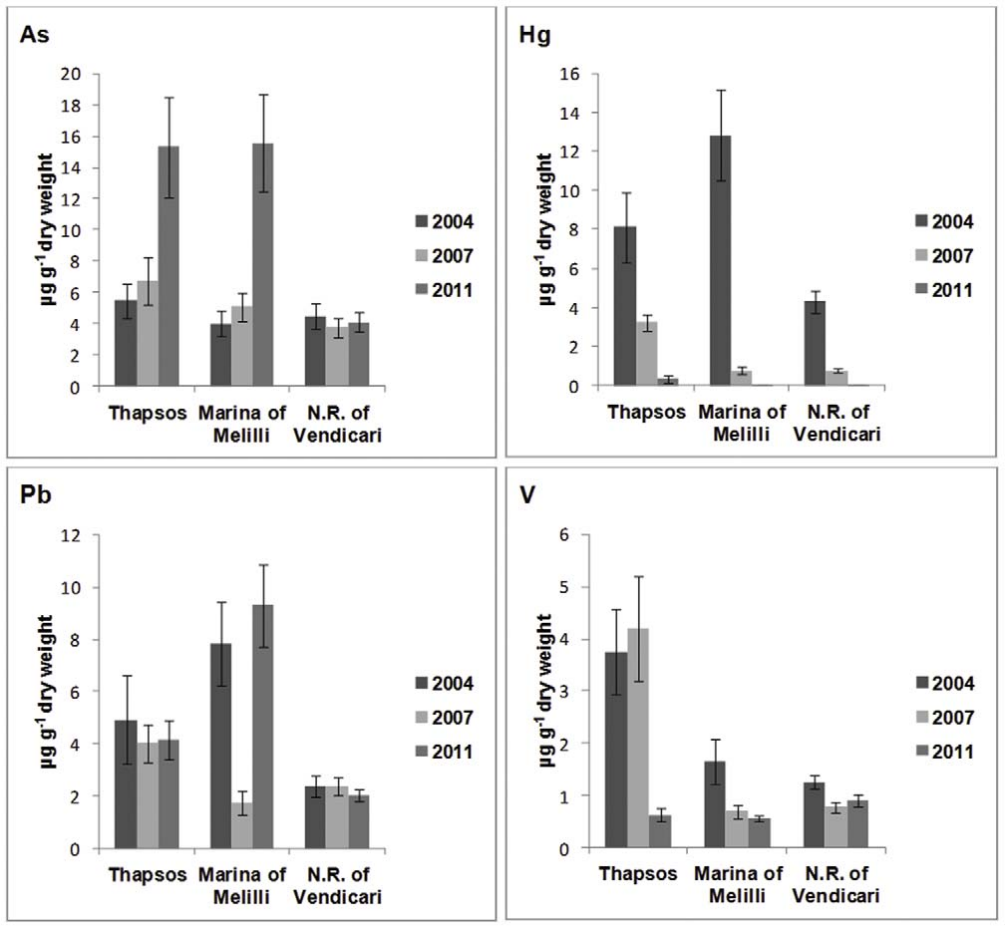
As, Hg, Pb and V content (μg g^−1^ dry weight) in the different samples.

The metals concentrations determined in the hepatopancreas are reported in Table 1. The content of heavy metals was generally higher in the animals collected from the polluted sites compared to those from the Vendicari Natural Reserve and, moreover, it was significantly different in relation to the year in which sampling was done. In the animals collected in 2004 from the two polluted sites (Table 1) the most significant data were those on the content of Hg and, to a lesser extent, of Pb, As and V,. In particular, the highest content of Hg – 12.84 μg g^−1^ dry weight – was found in animals from Marina di Melilli, whereas in those from Thapsos, which is located in front of the petrochemical plants responsible for the release in the sea of large quantities of waste containing Hg, the content of this metal was lower (8.13 μg g^−1^). However, the most striking feature was the presence of Hg (4.29 μg g^−1^) even in animals collected from the Vendicari Natural Reserve, site theoretically not involved in heavy metals pollution (Fig. 2). The animals collected in 2007 showed a Hg content still appreciable only in those collected from Thapsos, whereas those collected from Marina di Melilli and from the Vendicari Natural Reserve showed a very modest content of this metal (Table 1, Fig. 2). Seven years after the first sampling, the Hg content had decreased to very low levels in all animals collected from the three different sites (Table 1, Fig. 2).

**Table 1:** Heavy metal content (μg g^−1^ dry weight) of *Ligia italica* in the different samples.

THAPSOS MARINA OF MELILLI VENDICARI NATURAL RESERVE											
METALS	2004	2007	2011	2004	2007	2011	2004	2007		2011	
**Hg**	8.13±1.78	3.24±0.42	0.33±0.07	12.84±2.34	0.75±0.18	0.03±0.01	4.29±0.55		0.75±0.11		0.02±0.01
**As**	5.46±1.08	6.75±1.50	15.30±3.19	3.99±0.81	5.10±0.91	15.54±3.11	4.50±0.78	3.75±0.60		4.09±0.63	
**Cd**	0.24±0.07	0.36±0.11	1.05±0.22	0.27±0.09	0.22±0.06	1.26±0.17	0.33±0.07	0.25±0.04			0.22±0.07
**Cr**	0.27±0.09	1.68±0.27	1.41±0.22	0.18±0.05	0.67±0.14	1.50±0.31	0.21±0.04	1.01±0.13		0.39±0.06	
**Ni**	0.24±0.05	1.80±0.37	3.99±0.62	0.03±0.01	0.81±0.18	4.26±0.59	0.03±0.01	1.20±0.22		0.47±0.09	
**Pb**	4.92±1.70	4.02±0.73	4.17±0.73	7.83±1.62	1.74±0.45	9.30±1.56	2.37±0.40	2.37±0.34		2.04±0.22	
**V**	3.75±0.82	4.20±0.99	0.63±0.12	1.65±0.43	0.70±0.13	0.57±0.06	1.26±0.12	0.78±0.09		0.91±0.11	

Means ± SD.

The amount of other tested metals, such as Cd, Cr and Ni, was rather inconsistent, whereas low but appreciable concentrations of As, Pb and V, were detected in the animals collected from all sites (Table 1, Fig. 2). Statistically highly significant differences in the content of Hg, Pb and As were found in samples collected in the same year from the different sites, as well as in samples collected in different years from the same site (Table 2, Table 3).

**Table 2:** Kruskal-Wallis test.

YEARS	As	Hg	Pb	V
**2004**	2.76	7.20**	6.49*	5.96*
**2007**	5.07	5.53	6.49*	5.80*
**2011**	5.42	7.32	7.20**	1.69

Comparison of the different sites for the same year.

(H_0.05_  = 5.60 – *: p<0.05, H_0.01_  = 7.20 – **: p<0.01).

**Table 3:** Kruskal-Wallis test.

SITES	As	Hg	Pb	V
**Thapsos**	5.96*	7.20**	1.07	5.96*
**Marina of Melilli**	5.96*	7.26**	5.96*	5.42
**Vendicari N.R.**	0.80	7.26**	0.36	2.49

Comparison of the different years for the same site.

(H_0.05_  = 5.60 – *: p<0.05, H_0.01_  = 7.20 – **: p<0.01).

### Morphology and ultrastructure of hepatopancreas

**Figure 3:**
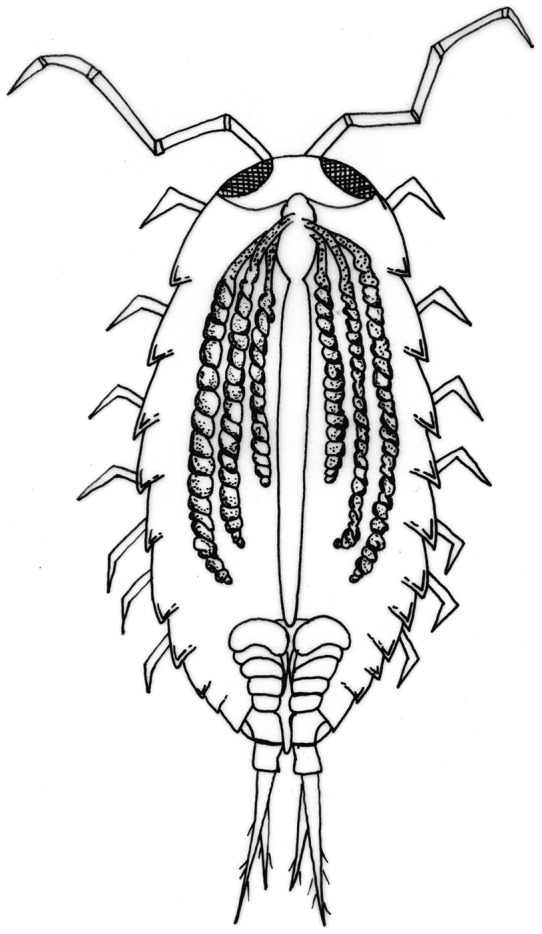
Schematic drawing of *in situ* hepatopancreas tubules of *Ligia italica*.

**Figure 4:**
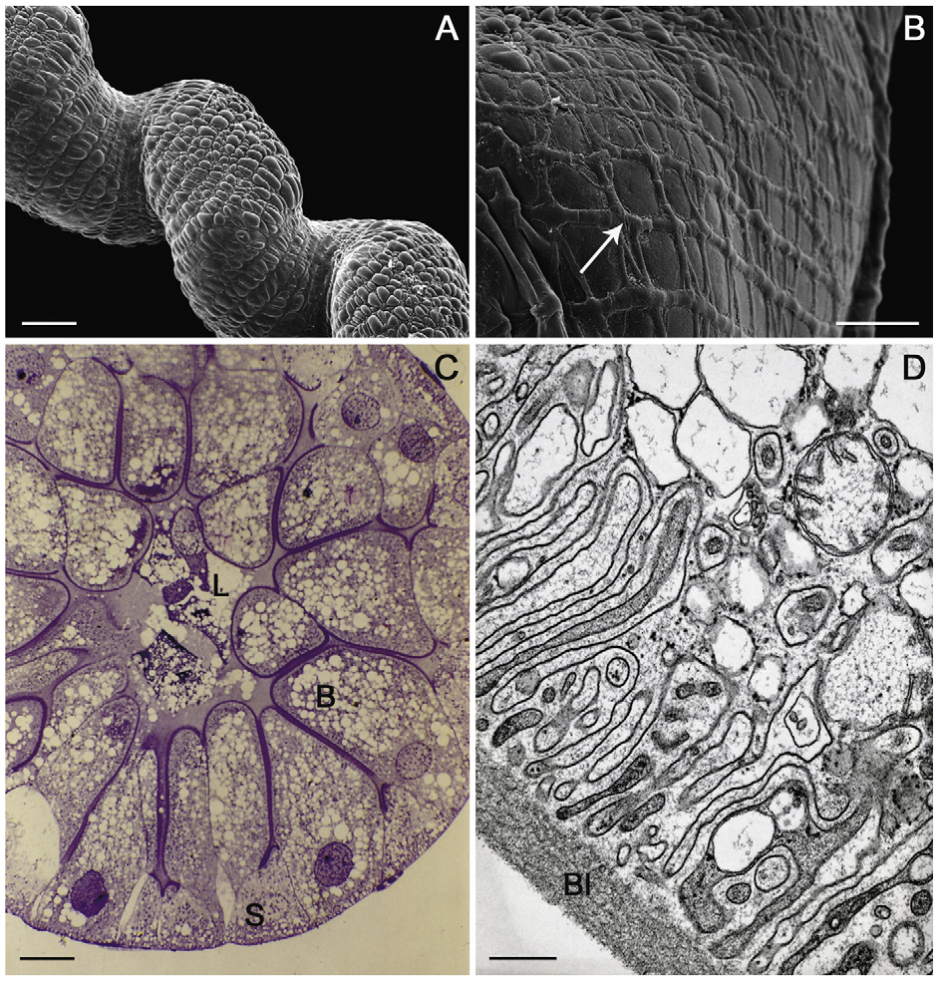
Tubule of the hepatopancreas of *Ligia italica* from Vendicari Natural Reserve. A–B: SEM, C: LM, D: TEM. A) The tubule shows a regular spiral trend and it is possible to observe the basal portions of S and B cells that protrude from its surface. B) A network of miocytes (arrow) surrounds the thin basal lamina that covers the surface of the tubules. C) Cross section of the proximal region of a tubule. B, dome-shaped large cells; S, small cells. The body of the B cells is filled with numerous lipid droplets. L, lumen of the organ (Semithin section; Sato and Shamoto stain). D) The basal plasmalemma of the B cells makes a typical membranous labyrinth characterized by a relevant number of tubular invaginations; Bl, basal lamina. Scale bar: A = 200 μm; B = 30 μm; C = 20 μm; D = 0.5 μm.

**Figure 5:**
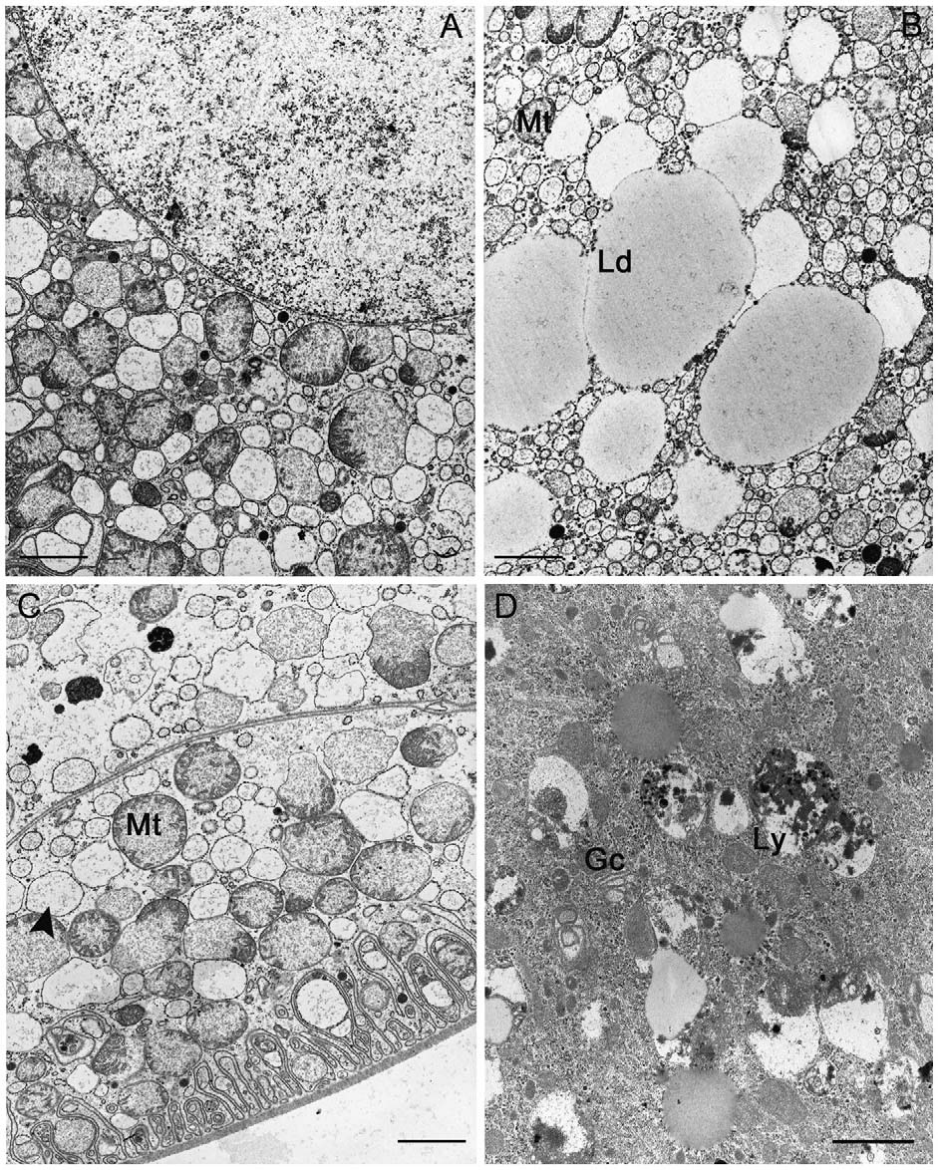
Tubule of the hepatopancreas of *Ligia italica* from Vendicari Natural Reserve. A–D TEM. A) Euchromatic nucleus of B cell. B) The cytoplasm of B cells contains abundant lipid droplets (Ld) of various sizes, mitochondria (Mt), RER elements and vesicles. C) Mitochondria (Mt) and RER elements (arrow head), particularly abundant in the subnuclear region of the B cells; Mt are swollen and show a great reduction of their cristae. D) Several saccules of the Golgi complex (Gc) and lysosomes (Ly) are present in the supranuclear region of the B cells. Scale bar: A–D  = 2 μm.

**Figure 6:**
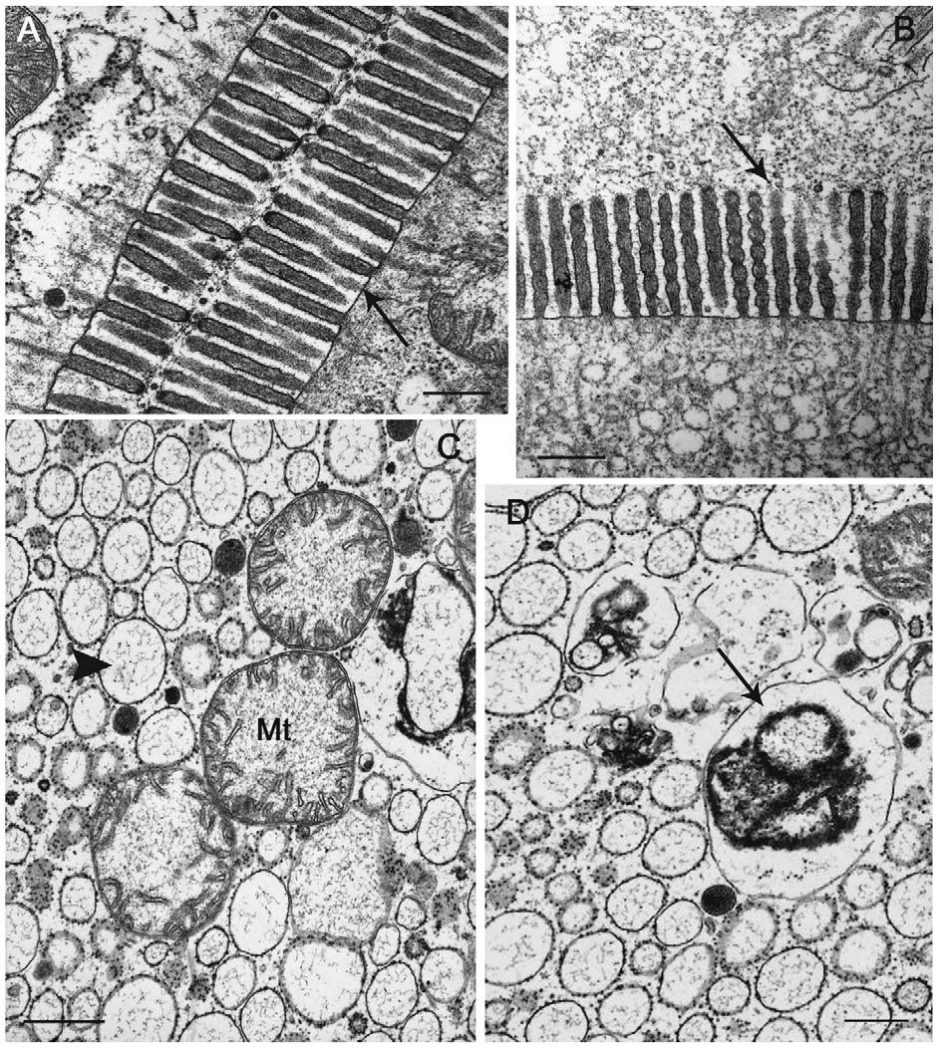
Tubule of the hepatopancreas of *Ligia italica* from Vendicari Natural Reserve. A–D TEM. A–B) Apical surface of B cells bearing a thick border of microvilli that in some cells undergo a progressive vesiculation (arrows). C) The cytoplasm of S cells is less dense and has a well developed rough endoplasmic reticulum in the form of rounded vesicles of various sizes (arrow head); also in the S cells the mitochondria (Mt) are swollen and with a clear reduction of their cristae. D) In the cytoplasm of S cells numerous vesicles (arrow) with a heterogeneous content, most likely of lysosomal derivation, are present. Scale bar: A, B = 0.5 μm; C = 1 μm; D = 1.5 μm.

In the animals collected from the Vendicari Natural Reserve in 2004, the hepatopancreas showed morphological and ultrastructural features very similar to those already known from other oniscidean isopods; however, while in the terrestrial isopods the hepatopancreas consists of two pairs of spiral blind-ending tubules that run adjacent to the hindgut throughout the length of the pereion, in the *L. italica*, as in the marine isopods, a third pair of shorter and thinner tubules is present (Figs. 3 and 4A). The histological structure of the tubules is very simple and almost homogeneous throughout their length with exception of the proximal and distal regions; their wall consists of a monolayered epithelium that lies on a thin basal lamina surrounded by a net of myocites (Fig. 4B). As in other species so far studied, two types of cells are present even in the epithelium of *L. italica*: the large basophilic cells (B cells) and the small cells (S cells), that alternate almost regularly in the organ cross sections (Fig. 4C). The B cells are dome-shaped and their apical portions protrude considerably into the organ's lumen (Fig. 4C). The wide basal plasmalemma of the B cells makes a typical membranous labyrinth, characterized by a relevant number of tubular invaginations (Fig. 4D). The large nucleus of the B cells is mostly located in a basal position; in some cells it is euchromatic, in others it shows a more condensed chromatin (Figs. 4C and 5A). The cytoplasm is slightly dense and contains abundant lipid droplets of various sizes, rough reticuloendothelial elements (RER) and vesicles in addition to numerous free ribosomes and glycogen granules (Fig. 5B). The mitochondria are numerous and prevalently localized in the subnuclear region (Fig. 5A); most of them appear swollen and show a great reduction of their cristae (Fig. 5C). The rough endoplasmic reticulum is extended above and below the nucleus; in some B cells it is arranged in small cisternae that show some fenestrations while in most of them it is formed by small vesicles or large vacuoles (Figs. 5B–C). The apical regions of the B cells show a thick border of microvilli (Fig. 6A) that in some cells undergo vesiculation and subsequent disorganization (Fig. 6B). The S cells are wedge-shaped and are much lower than the B cells; their lateral surface is closely adjacent to that of the B cells, so, only a small extent of their apical surface overlooks the lumen of the hepatopancreas (Fig. 4C). Many of the characteristics of the S cells are comparable to those of the B cells but their cytoplasm is less dense and the nucleus has sometimes an irregular shape. The RER is well developed and consists mostly of rounded vesicles (Fig. 6C); golgian structures and lipid droplets are rarely observed. Small and rounded mitochondria showing a reduction of their cristae are scattered in the cytoplasm as small vesicles with an electron-dense content (Fig. 6C). The S cells contain, moreover, some vesicles that store metals and others with a heterogeneous content, most likely of lysosomal derivation (Fig. 6D).

**Figure 7:**
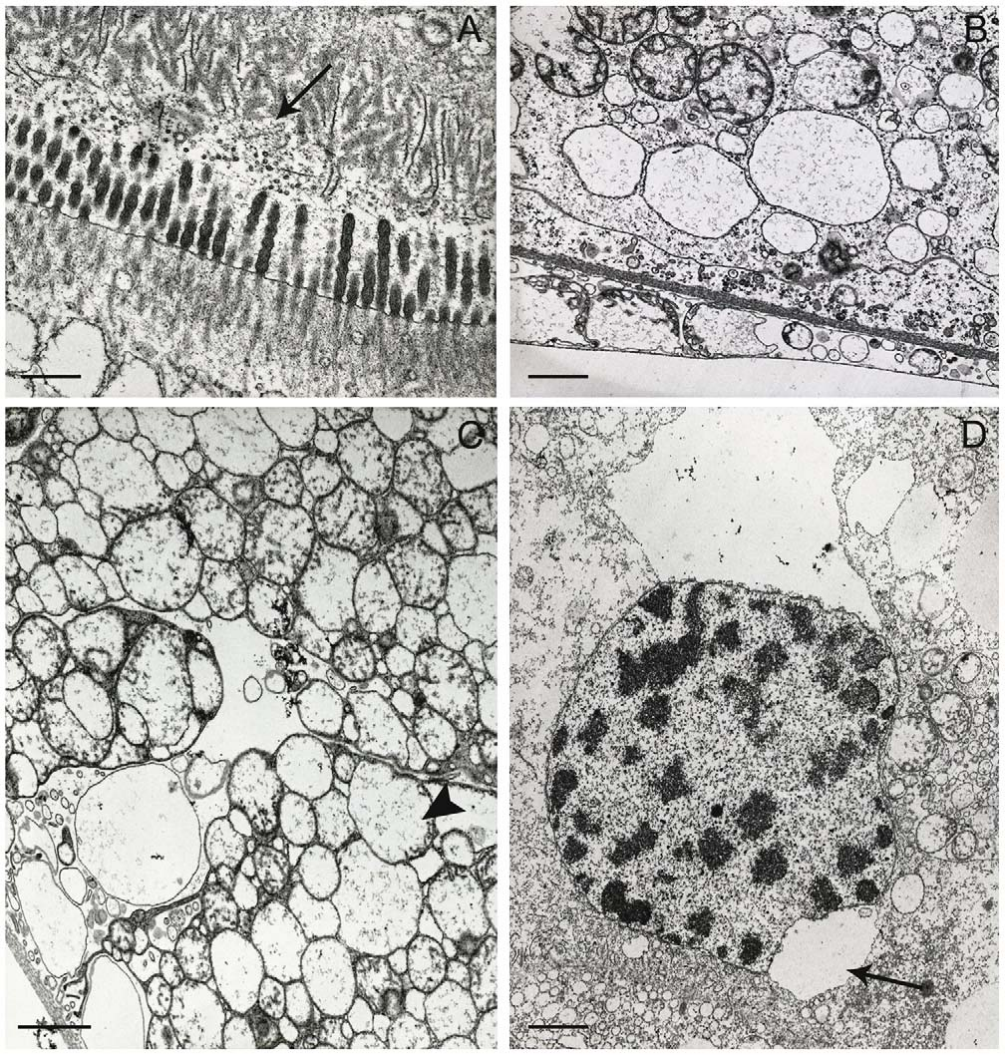
Tubule of the hepatopancreas of *Ligia italica* from the two polluted sites. A–D TEM. A) Most of the B and S cells show a marked alteration of the microvillous border that, in some cells, undergoes a total disorganization (arrow). B) The B cells show a partial or total disappearance of the basal membranous labyrinth. C) The mitochondria of B cells undergo a more marked swelling and are also almost completely devoid of cristae; many show a frank cavitation of matrix (arrow head). D) The nuclei of B cells exhibit a considerable reduction of their volume, consequent to a massive condensation of chromatin, sometimes associated with some large dilatations (arrow) of their perinuclear cisterna. Scale bar: A = 0.5 μm; B = 1 μm; C = 1.5 μm; D = 2 μm.

In the specimens of *L. italica* collected in 2004 from the two polluted sites, the hepatopancreas does not show considerable morphological modifications but rather it undergoes more marked alterations of the cellular ultrastructure that have affected the majority of cells present along the entire wall of the organ. Some of these alterations are common to the two types of cells, some are more specific. The most important alterations common to both types of cells relate to:

- damage of the microvillous border; this alteration does not affect all cells equally as in some of them the microvilli retain an aspect almost normal, in others they show an irregular profile like “sausage”, in others they undergo a process of vesiculation that results in their complete disorganization (Fig. 7A);
- partial or total disappearance in many cells of the basal membranous labyrinth (Fig. 7B);
- a more marked swelling of the mitochondria that are also almost completely devoid of cristae and frequently present an open cavitation of the matrix (Fig. 7C);
- a notable reduction of the nuclear volume consequent to a massive condensation of chromatine, sometimes associated to some large dilatations of perinuclear cisterna (Fig. 7D).

**Figure 8:**
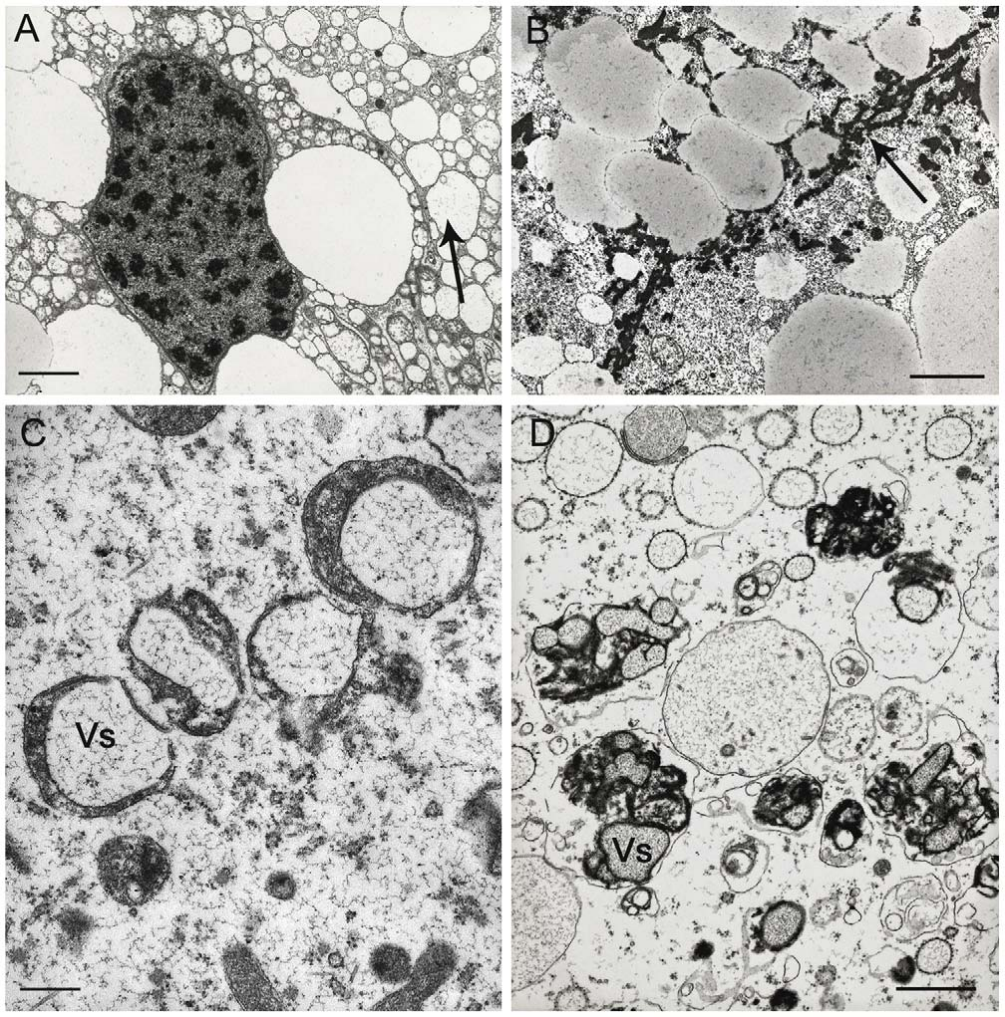
Tubule of the hepatopancreas of *Ligia italica* from the two polluted sites. A–D TEM. A) In many B cells the rough endoplasmic reticulum undergoes a marked swelling and vesiculation (arrow); large vacuoles (Lv), probably derived from the rough endoplasmic reticulum, are present around the nucleus. B) The lipid droplets of B cells are surrounded by scattered accumulations of electron-dense material that in some cells are particularly abundant (arrow). C) In many S cells the number of vesicles of heavy metals accumulation increases moderately. D) The major significant ultrastructural alteration concerning the S cells is, however, represented by the presence of numerous vesicles containing heterogeneous electron-dense material (Vs), that are most likely of lysosomal derivation. Scale bar: A, B = 2 μm; C = 0.5 μm; D = 1 μm.

Other alterations regarding B cells are:

- further increased cytoplasmic density;
- considerable swelling and vesiculation of the RER; large vacuoles, probably derived from the RER, are present around the nucleus in some B cells (Fig. 8A);
- presence in their cytoplasm, near the lipid droplets, of small scattered accumulations of electron-dense material that in some cells become much more large and abundant (Fig. 8B).

The major ultrastructural alterations concerning the S cells are represented primarily by an increase in the number of vesicles that accumulate heavy metals (Fig. 8C) and of other vesicles containing an heterogeneous electron-dense material, most likely of lysosomal derivation (Fig. 8D).

In the specimens collected in the subsequent samplings the ultrastructural alterations highlighted in the hepatopancreas were quite irrelevant.

## Discussion

Our research, based on a series of sampling carried out in two different sites of Eastern Sicily, has shown that three years after the discharge into the sea of a massive amount of Hg by a petrochemical industry located in the immediate vicinity of the study areas, a significant amount of this metal as well as modest concentrations of other metals, such as As, Pb, and V, were still present in tissues of *Ligia italica*, an oniscidean isopod, frequent and abundant in the rocky supralittoral zone of those sea coasts. Data get more significant value if we consider that Hg, after its discharge into the marine environment, is in part linked to suspended sediments (therefore it is temporarily buried) and then undergoes various processes of transformation and biodegradation including chemical modifications [33]–[37], so that its concentration in the upper layers of marine waters (that come into contact with *L. italica*) is significantly reduced. Surprisingly, a fair amount of Hg was also found in some *L. italica* collected from the Vendicari Natural Reserve, the environment chosen as a control area (because presumably free from Hg pollution), located about 30 miles away from the industrial plant responsible for the Hg pollution. This result would indicate that the discharge could propagate, before its immobilization, even at a considerable distance from the release site thanks to the sea currents and the atmospheric spread, as well as to the marine sediments of the primarily polluted site made up of coarse-grained material that could have scavenged only a limited portion of the total Hg [38], thus helping the spreading of the Hg pollution.

In animals collected from the same sites, three and seven years after the first sampling respectively, concentrations of As, Cd, Cr, Ni, Pb and V showed more or less modest changes, probably related to their variable discharge into the marine environment from chemical plants present in the area under examination; the amount of Hg found in the animals of the two polluted sites has, instead, undergone a progressive and marked decrease, until reaching values close to zero in the samples from Marina di Melilli and from the Vendicari Natural Reserve. This would suggest that industries have not performed a further discharge of waste containing Hg and/or that the significant amount of Hg released into the sea in 2001 and in previous years has been subject of an outflow represented, in descending order, by the sedimentation of particles containing Hg, by the offshore exports and by the dilution in the atmosphere as a result of volatilization [35].

The ultrastructural investigations carried out after the first sampling on the hepatopancreas of animals collected from the two heavily polluted sites, showed a considerable set of cellular alterations that affected both cell types present in the wall of the organ; most probably, these alterations are ascribable to the bioaccumulation of mercury even if cannot be entirely excluded an additional influence resulted from the contemporaneous presence of other metals such as As, Pb and V. Some of these cellular alterations, although less marked and more sporadic, have also been observed in the hepatopancreas of the animals collected from the control area of the Vendicari Natural Reserve. These alterations have not equally involved the entire wall of the organ but have variably involved a more or less relevant number of both types of its epithelial cells. The most significant cellular effect was certainly represented by the disorganization of the microvillous border that is, undoubtedly, the first target of the action exerted by the metal. Some B and S cells have only shown a moderate decrease in the number of microvilli that appear shorter and slightly deformed; in other cells the microvilli undergo a progressive vesiculation or total destruction associated with the disappearance of their actin cytoskeleton and of the below terminal net. A similar effect has already been described by Köhler et al. [28] for the hepatopancreas of *Porcellio scaber* contaminated with high concentrations of Cd, Pb an Zn and represents, therefore, the result of a mechanism common to many heavy metals; however, the same changes have also been reported by Storch e Lehnert-Moritz [39] for the hepatopancreas of animals of *Ligia oceanica* subjected to a prolonged starvation period. The reduced functionality of the microvillous border has an obvious negative effect on the absorption of nutrients by the hepatopancreas [40].

Another main target of the metal is the labyrinth formed by the plasma membrane in the basal region that undergoes a remarkable reduction in most of the B cells. In their research on the toxic effect of Hg in *Crangon crangon*, Andersen and Baatrup [41] reported that most of the metal accumulated in the hepatopancreas was absorbed from the haemolymph; this finding would explain the severe damage of the plasma membrane also in the basal region of the B cells. The damage induced by Hg on the basal membranous labyrinth do not seem to be induced by other heavy metals; Köhler et al. [28], in fact, in specimens of *P. scaber* contaminated with high concentrations of Cd and Pb never observed a significant alteration of the basal labyrinth.

Other alterations consequential to the Hg accumulation involve:

- the RER that undergoes a more or less pronounced fragmentation and consequent swelling and vesiculation in many B and S cells;
- the mitochondria, that show in a large extent a significant swelling and a marked reduction or even a total disappearance of their cristae;
- a decay of lipid droplets often showing at the periphery the presence of clusters of electron-dense material probably corresponding to lipofuscin;
- a marked condensation of nuclear chromatine often associated with one or more large dilatations of perinuclear cisterna;
- an increased number of vesicles of lysosomal derivation filled with heterogeneous electron-dense materialIn other organisms too, lysosomes have been identified as the major site of Hg accumulation [42]–[44].

Many of the effects that we have observed in *L. italica* are largely similar to those described in *P. scaber* by Köhler et al. [28] who argue that these ultrastructural alterations could be used as biomarkers for monitoring the subchronic exposure to the heavy metals present in the environment. Nevertheless, as stated by the same aforementioned authors, a pattern of similar alterations were also found in the hepatopancreas of animals of different species of oniscideans subjected to prolonged starvation [39], [42]–[47]; therefore, in our opinion, the ultrastructural effects of Hg or other heavy metals accumulation could be used as biomarkers only if supported by a qualitative and quantitative analysis of the heavy metals content.

As a conclusion, based on what we said in the introduction about a bioindicator, we can say that *L. italica* is a good indicator for Hg pollution, as it is accessible, because easily available and easy to sample, and it has a very accessible threshold of analytical detection with standard techniques of analysis. *L. italica* is suitable from the bio-ecological point of view because it shows a wide distribution in the study area; its identification is easy, and there is an adequate knowledge on its anatomy, physiology and ecology; moreover, it has a sufficient life cycle and, even more important, it has poor mobility and it is easily available in all seasons. Our results, also, show its reliability and representativeness.

Despite all these strong points we have to highlight that in our study there are still some important weak points that are the lack of data about the content of the metal in the water and soil of the study area, and, in addition, the little knowledge about genetic of *L. italica* that not disclose its possible allopatric differentiation, therefore they lack information about its adaptability to constantly changing environmental conditions. These weak points, however, are easily overcome with appropriate studies, that we are planning in a near future.
